# Esophageal Exclusion as a Damage-Control Strategy for Acute Esophageal Necrosis Secondary to Ruptured Stanford Type B Aortic Dissection: A Case Report

**DOI:** 10.7759/cureus.115196

**Published:** 2026-08-25

**Authors:** Ruana Rodriguez Rueda, Nikolaos Koliakos, Freddy Mboti

**Affiliations:** 1 Department of Abdominal Surgery, Erasme Hospital, Université Libre de Bruxelles, Brussels, BEL; 2 Department of Digestive, Thoracic, and Laparoscopic Surgery, Brugmann University Hospital, Brussels, BEL

**Keywords:** acute esophageal necrosis (aen), aortic-esopaheal fistula, compartimental thoracic syndrome, damage-control surgery, endovascular aneurysm repair, esophagostomy, temporary esophageal exclusion, thoracic endovascular aortic repair, total esophagectomy, type b acute aortic dissection

## Abstract

Acute esophageal necrosis (AEN) is a rare and life-threatening condition most commonly associated with systemic hypoperfusion and critical illness. Aortic-related AEN is exceptionally uncommon and may result from direct mediastinal compression and vascular compromise rather than generalized hemodynamic insufficiency. We report the case of a 77-year-old man presenting with a Stanford type B aortic dissection associated with aneurysmal degeneration complicated by rupture. The patient presented with progressive dysphagia, back pain, and hemoptysis. Computed tomography angiography demonstrated a large mediastinal hematoma causing marked compression of the thoracic esophagus. Following emergency thoracic endovascular aortic repair (TEVAR), the patient experienced a prolonged intensive care stay complicated by acute respiratory distress syndrome (ARDS) and pulmonary infection. Persistent dysphagia and subsequent gastrointestinal bleeding prompted endoscopic investigation, which revealed progressive esophageal injury evolving from severe erosive esophagitis to transmural necrosis with multiple fistulas and ultimately an aortoesophageal fistula. Given the patient’s severe malnutrition, prolonged illness, and mediastinal contamination, esophagectomy was considered prohibitively high risk. Esophageal exclusion and feeding jejunostomy were, therefore, performed as a damage-control strategy to achieve source control. Despite successful diversion of the esophageal stream, the patient died from progressive systemic complications. This case highlights the diagnostic challenge of aortic-related esophageal ischemia and emphasizes that persistent dysphagia, gastrointestinal bleeding, or unexplained inflammatory deterioration following thoracic aortic rupture, dissection, or TEVAR should prompt consideration of esophageal involvement. It also supports the role of esophageal exclusion as a damage-control option in selected high-risk patients who are unsuitable for definitive esophagectomy.

## Introduction

Acute esophageal necrosis (AEN), also known as “black esophagus,” is a rare but life-threatening condition with a reported incidence ranging from 0.01% to 0.28% and is characterized by circumferential ischemic injury of the esophageal mucosa [1]. The condition most commonly occurs in critically ill patients with systemic hypoperfusion, cardiovascular disease, malnutrition, renal dysfunction, or other conditions associated with impaired tissue perfusion [1,2]. Conservative management consisting of intravenous fluid resuscitation, blood transfusion when indicated, acid suppression therapy, and nutritional support results in clinical resolution in a substantial proportion of cases [2].

A distinct and particularly aggressive form of AEN may occur in association with thoracic aortic pathology. In patients with acute aortic rupture or dissection, the development of a large mediastinal hematoma can produce direct extrinsic compression of the esophagus and compromise regional arterial blood supply, resulting in severe localized ischemic injury [3-5]. Unlike the more common hemodynamic form of AEN, tissue necrosis in this setting results primarily from mechanical compression and vascular compromise rather than generalized hypoperfusion. 

Because early manifestations are often nonspecific, diagnosis is frequently delayed until transmural necrosis, perforation, or aorto-esophageal fistula develops, resulting in poor outcomes [4-6]. Owing to the rarity of this condition, the optimal timing of diagnosis and surgical management remains poorly defined, with published evidence limited to isolated case reports and small case series. We report a case of progressive esophageal ischemia and transmural necrosis following a ruptured Stanford type B aortic dissection treated with TEVAR, managed with esophageal exclusion as a damage-control strategy. In addition, we review the available literature on aortic-related esophageal necrosis, with particular emphasis on the mechanisms of injury, diagnostic challenges, and surgical management. 

## Case presentation

**Figure 1:**
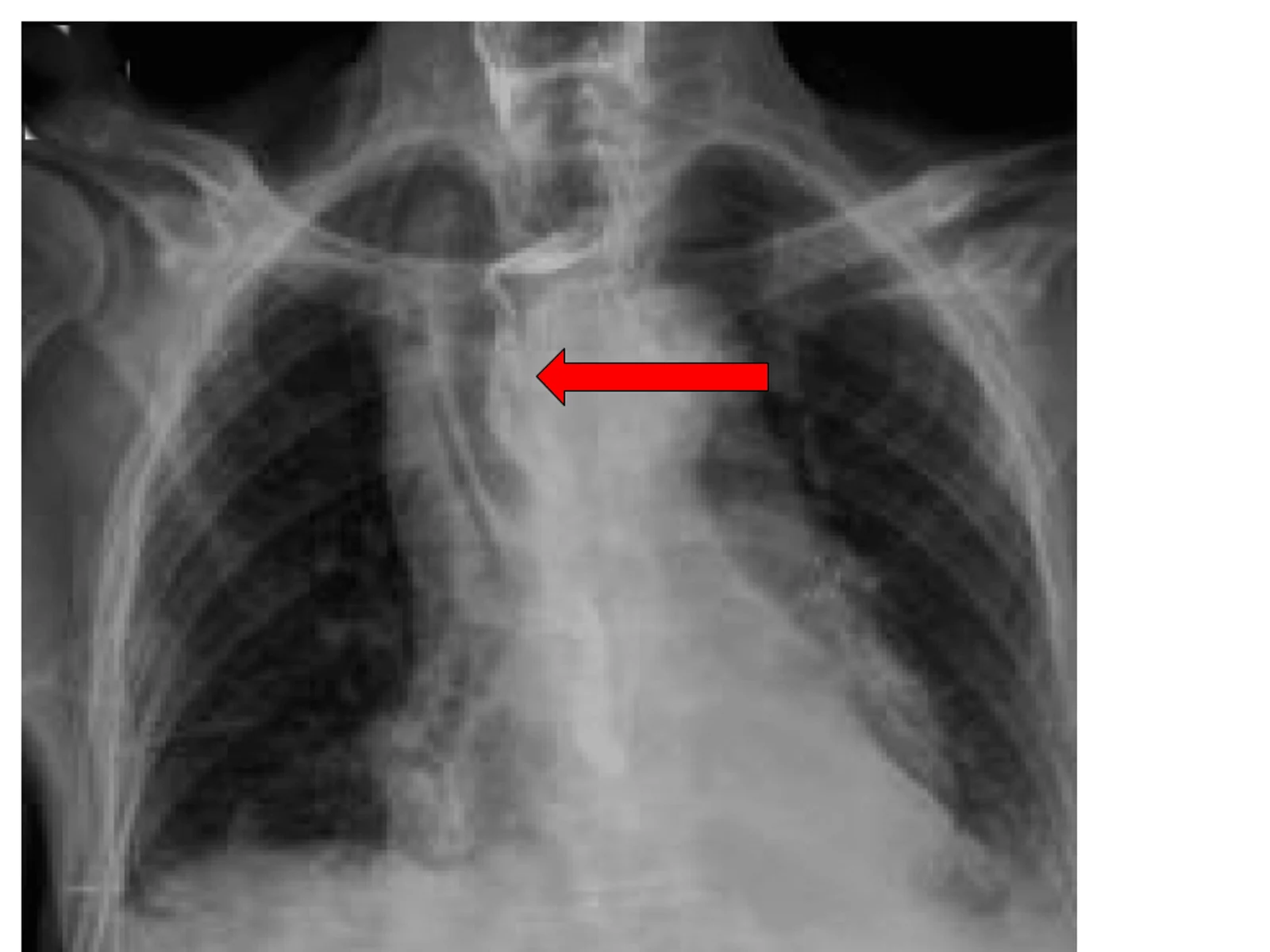
Frontal chest radiograph of the esophagus The esophagus is displaced to the right by the mediastinal hematoma

A 77-year-old man with a medical history of hypertension, atrial fibrillation, receiving anticoagulation therapy, and a known descending thoracic aortic aneurysm presented to the emergency department with a one-week history of progressive back pain, dysphagia, and hemoptysis. On admission, he appeared cachectic and pale. Vital signs demonstrated hemodynamic instability with a mean blood pressure of 69 mmHg and a heart rate of 112 beats/min. Laboratory investigations revealed leukocytosis (14.57 × 10³/µL), elevated C-reactive protein (200 mg/L), severe coagulopathy (international normalized ratio (INR): >9), and acute kidney injury with an estimated glomerular filtration rate of 26 mL/min/1.73 m². A thoracic X-ray shows the esophageal deviation to the right side (Figure 1).

**Figure 2:**
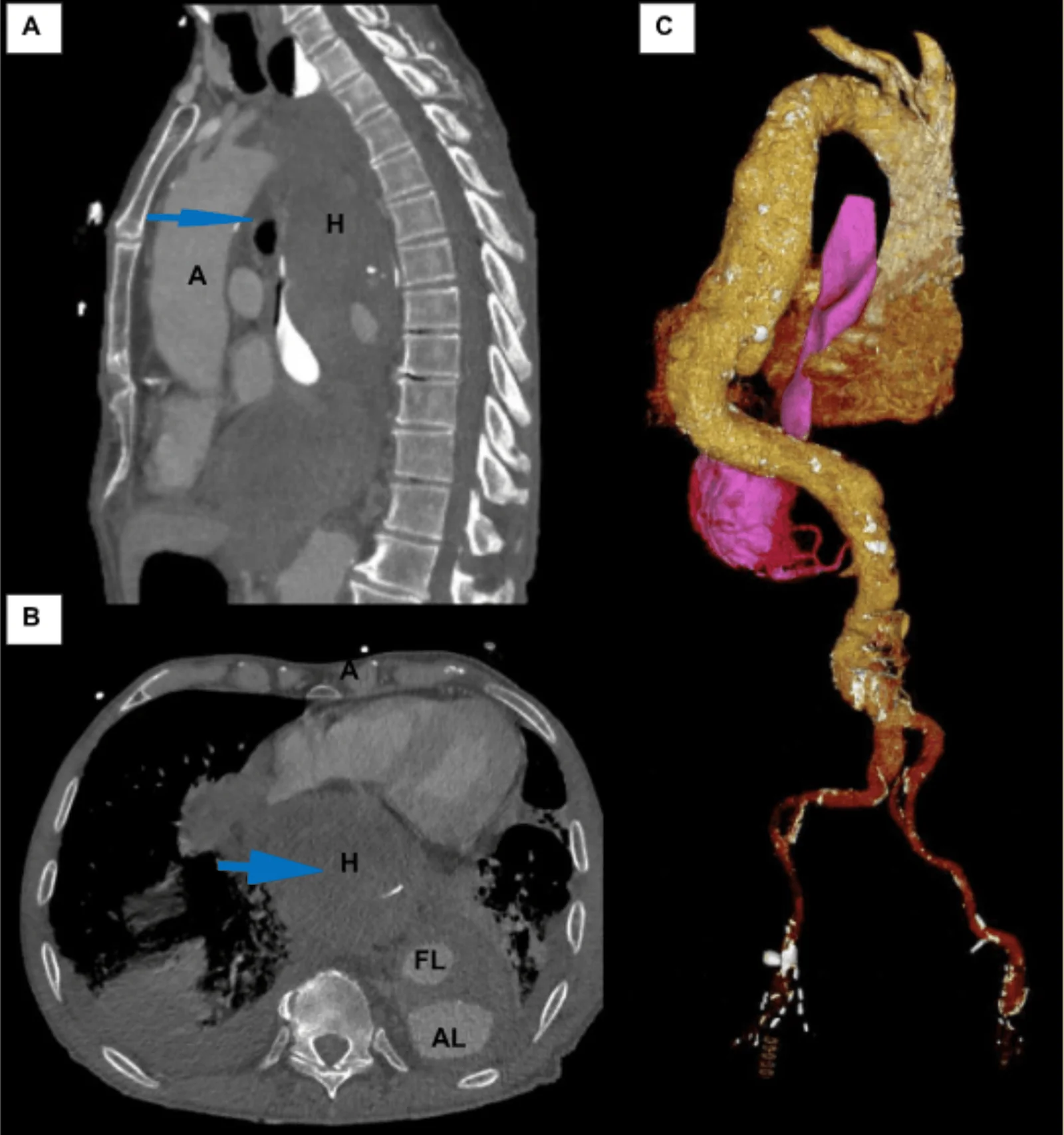
CT scan with gastrografin demonstrating thoracic hematoma and esophageal compression H: hematoma; A: aorta; H: hematoma; A: aorta; AL: aortic lumen; FL: false lumen (A) Sagittal reconstruction showing compression of the esophageal lumen by the mediastinal hematoma. (B) Axial reconstruction showing compression of the esophageal lumen. (C) Three-dimensional CT reconstruction of the aorta in a right posterior oblique view, with gastric contents opacified by contrast in pink

Computed tomography angiography demonstrated rupture of a descending thoracic aortic aneurysm with active extravasation and a large mediastinal hematoma measuring 67 × 64 mm, causing marked compression of the thoracic esophagus (Figures 2A-2C). The patient underwent emergency thoracic endovascular aortic repair (TEVAR) 24 hours after his admission; two 150-mm thoracic stent grafts were deployed, with the proximal graft positioned approximately 5 cm distal to the left subclavian artery and the distal graft extending to just above the celiac trunk.

The postoperative course was complicated by acute respiratory distress syndrome (ARDS), requiring prolonged intensive care management. Nutritional support proved challenging; consequently, a surgical gastrostomy was performed on hospital day 13. Episodes of atrial fibrillation were treated with amiodarone.

**Figure 3:**
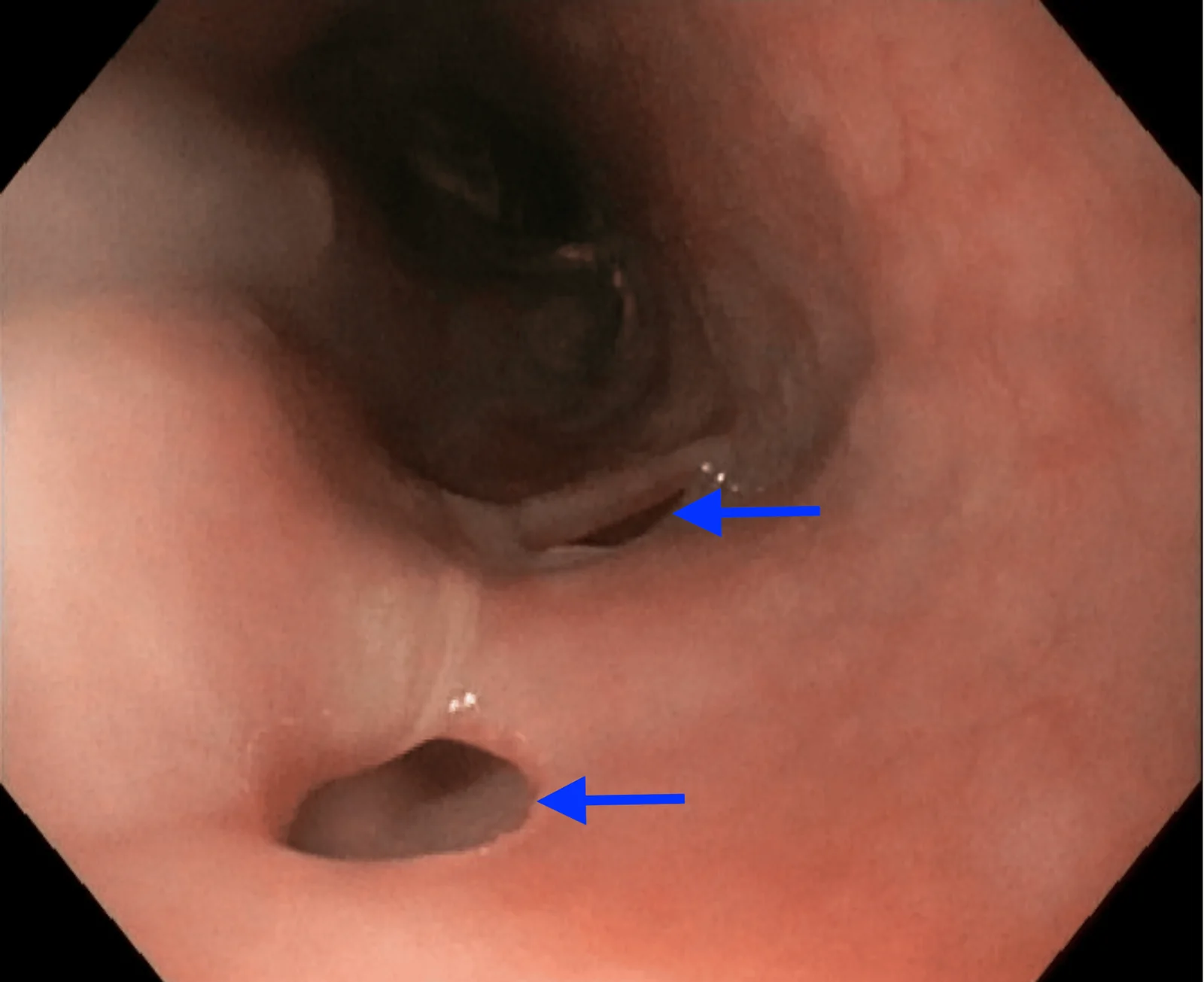
Endoscopic view showing two fistulous openings of the esophageal-mediastinal fistula

**Figure 4:**
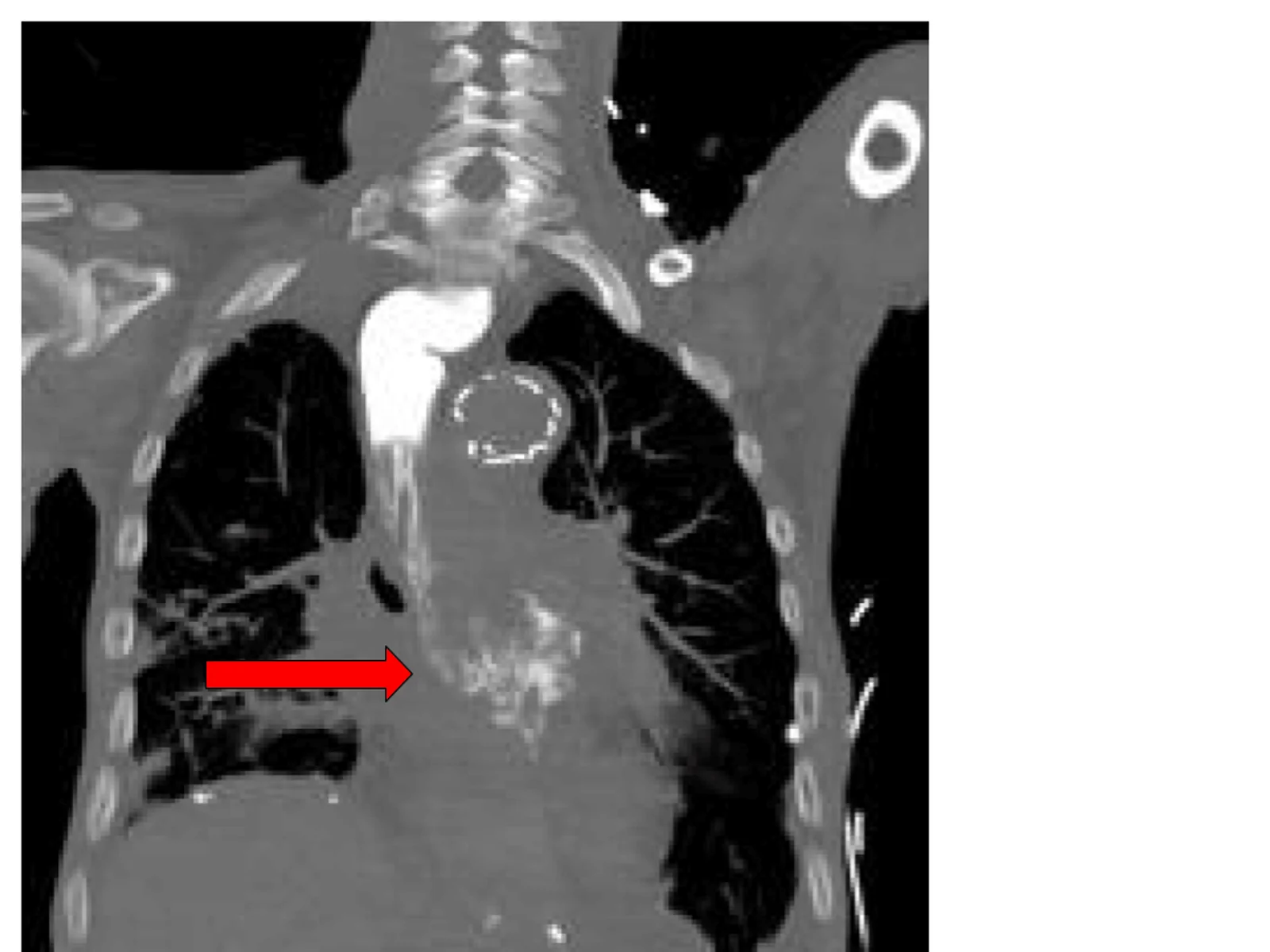
Coronal CT reconstruction after gastrografin administration

Following a prolonged stay in the intensive care unit and subsequent clinical stabilization, the patient was transferred to the Department of Visceral Surgery for further management and nutritional support. On hospital day 30, the patient developed melena, prompting esophagogastroduodenoscopy (EGD), which demonstrated erosive esophagitis, an intraluminal blood clot, a mucosal bridge within the thoracic esophagus (Figure 3), and local food impaction. Repeat EGD performed three days later (hospital day 33) revealed multiple fistulous defects, one of which allowed visualization of the right pulmonary parenchyma through the fistulous tract. Computed tomography performed confirmed an aortoesophageal fistula, demonstrating leakage of orally administered contrast into the mediastinal cavity and surrounding hematoma (Figure 4). A further endoscopic examination identified an approximately 10 cm segment of extensive esophageal destruction that was considered unsuitable for endoscopic stent coverage.

**Figure 5:**
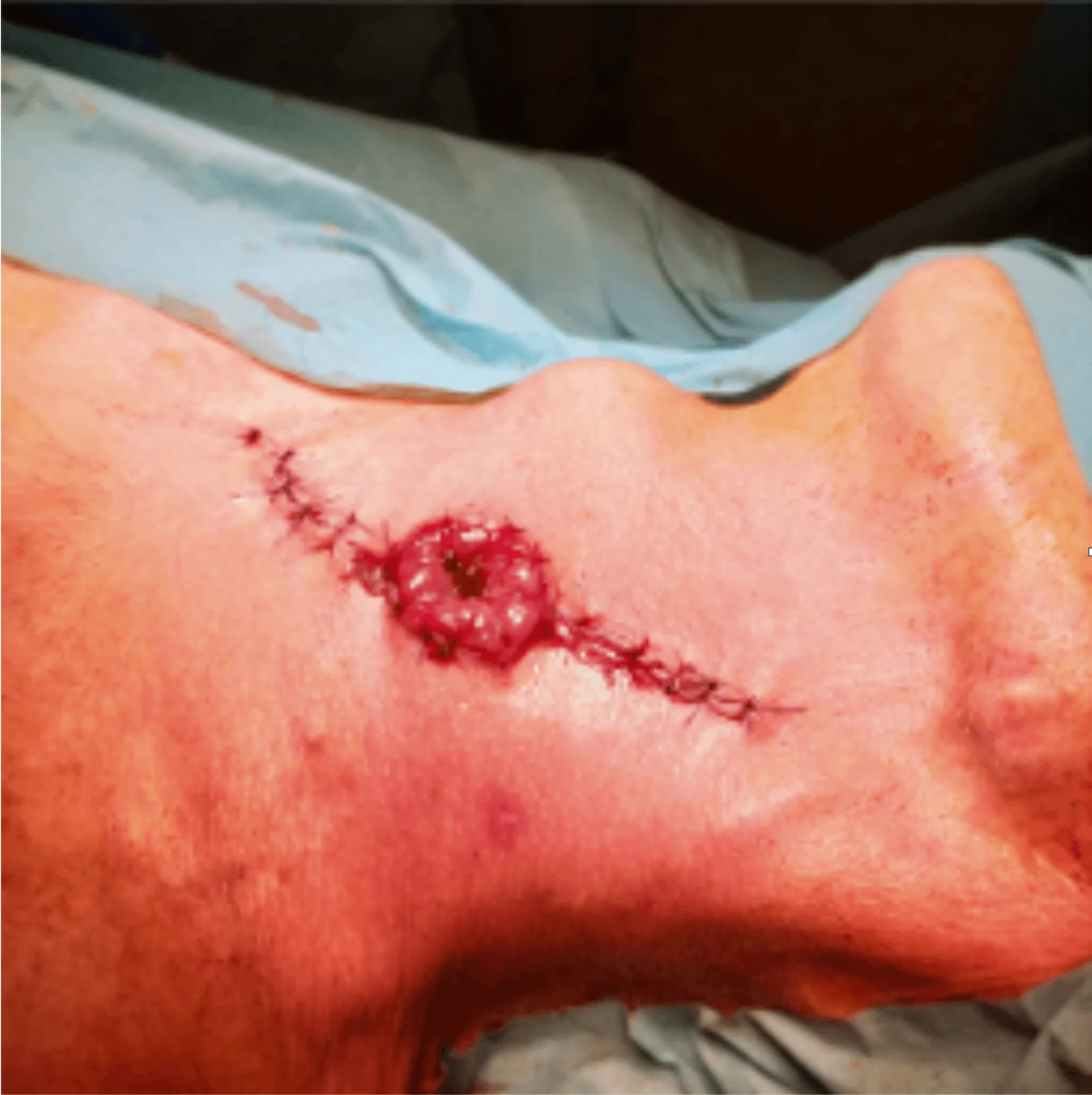
Esophageal exclusion: esophagostomy made in the left cervicolateral position

Given the patient’s frailty, severe malnutrition, and mediastinitis, and the unsuccessful attempt at endoscopic stenting, definitive surgery was initially deferred to allow clinical and nutritional optimization; esophageal exclusion was performed on hospital day 65 by means of cervical esophagostomy (Figure 5) and feeding jejunostomy. However, despite intensive supportive treatment, he developed acute respiratory deterioration on hospital day 72 followed by cardiac arrest. Resuscitative efforts were unsuccessful, and the patient passed away. No postmortem examination was performed.

## Discussion

AEN is an uncommon but potentially fatal condition characterized by ischemic injury of the esophageal wall. Most reported cases occur in critically ill patients with systemic hypoperfusion and multiple comorbidities, where a combination of vascular insufficiency, impaired mucosal defense mechanisms, and gastric reflux contributes to tissue injury [1,2]. In contrast, the present case illustrates a much rarer mechanism of esophageal necrosis secondary to thoracic aortic pathology.

In our case presentation, rupture of a descending thoracic aortic aneurysm resulted in the formation of a large mediastinal hematoma that compressed the esophagus and likely compromised its vascular supply. Unlike the classical hemodynamic form of AEN, ischemia in this setting appears to result primarily from local mechanical compression and disruption of regional arterial perfusion. Similar mechanisms have been described following thoracic aortic rupture, aortic dissection, thoracoabdominal aneurysm repair, and TEVAR [4,5,7]. De Praetere et al. suggested that long-segment stent grafting may compromise esophageal perfusion through interruption of segmental arterial supply, while Koizumi et al. reported esophageal necrosis after endovascular repair of a ruptured aortic dissection, highlighting the vulnerability of the esophagus to ischemic injury in the setting of acute aortic disease [5,7].

The clinical presentation of aortic-related esophageal ischemia can be particularly challenging. Progressive dysphagia associated with persistent inflammatory markers, gastrointestinal bleeding, recurrent infection, or signs of sepsis should raise suspicion for evolving esophageal injury and possible progression toward necrosis or perforation [2-4,6]. Several reports have highlighted that esophageal complications are often referred late because early symptoms are nonspecific and are frequently overshadowed by the severity of the underlying aortic disease and associated critical care complications (Table 1) [5-18]. In the present case, clinical attention was initially directed toward treatment of the ruptured thoracic aneurysm and subsequent respiratory complications, including ARDS and pulmonary infection. Definitive diagnosis was only established after the occurrence of melena and endoscopic investigation. The ischemia progression in this patient highlights the dynamic nature of ischemic esophageal injury. Initial endoscopy demonstrated erosive esophagitis, whereas only three days later it revealed multiple fistulous tracts. This rapid deterioration suggests that esophageal damage may continue to evolve despite successful aortic repair and underlines the importance of maintaining a high index of suspicion in patients with persistent dysphagia or unexplained inflammatory worsening following TEVAR [6,7]. Such delays in diagnosis remain a major challenge and are associated with significant morbidity and mortality [19].

**Table 1:** Summary of reported cases of aortic-related esophageal necrosis TEVAR: thoracic endovascular aortic repair

Author	Year	Age	Gender	Diagnosis: aortic entity	Days to diagnosis	Treatment	Mortality
Watanabe et al. [8]	1999	76	M	Ruptured dissection type B Stanford	8	Segmental esophagectomy	1
Minatoya et al. [9]	2000	66	F	Aortic dissection type A + B Stanford	41	None	1
Park et al. [10]	2004	47	M	Traumatic aortic rupture	7	Esophageal exclusion	1
Kaneda et al. [11]	2005	80	M	Aortic dissection type B Stanford	3	None(wish of the family)	1
Porcu et al. [12]	2005	59	M	Leak after TEVAR for Stanford B	30	Kher drain	1
Rascanu et al. [13]	2009	74	F	Ruptured dissection type B Stanford	14	Esophageal stent	1
De Praetere et al. [5]	2010	72	M	Ruptured thoracoabdominal aortic aneurysm	20	None (wish of the family)	1
Seto et al. [14]	2014	67	M	Ruptured dissection type B Stanford	20	Esophageal exclusion	1
Tobisch et al. [15]	2014	65	F	Ruptured dissection type B Stanford	15	Esophagectomy	0
Iuamoto et al. [16]	2015	53	M	Aortic dissection type B Stanford	6	Esophagectomy	0
Abou-Al-Shaar et al. [17]	2016	56	M	Ruptured thoracoabdominal aortic aneurysm	14	Esophageal exclusion	0
Joubert et al. [18]	2016	66	M	Aortic dissection type B Stanford	3	Esophagectomy	0
Koizumi et al. [7]	2017	78	M	Ruptured dissection type B Stanford	9	Died before intervention	1
Chacón et al. [6]	2022	46	M	Aortic dissection type B Stanford	1	Esophagectomy	1

Management of transmural esophageal necrosis after aortic aneurysm rupture remains poorly standardized because most available evidence consists of case reports, registry data, and small retrospective series [5,7,11]. Treatment must address both the aortic pathology and the esophageal source of contamination [17,20]. Definitive esophagectomy with mediastinal debridement is generally considered the most radical treatment option and may offer the best chance of long-term survival; however, it is associated with substantial morbidity and mortality, particularly in physiologically compromised individuals [21,22]. In the present case, prolonged critical illness, severe malnutrition, persistent inflammatory syndrome, and extensive mediastinal contamination rendered major thoracic surgery prohibitively high risk. For these reasons, esophageal exclusion by cervical esophagostomy and feeding jejunostomy was selected as a damage-control strategy [23]. This approach is consistent with prior reports describing esophageal diversion or exclusion in complex perforations and with literature emphasizing the need for individualized, multidisciplinary decision-making according to the extent of contamination and the patient’s physiological reserve [23]. Despite this intervention, the prognosis remains poor once transmural necrosis, perforation, or aorto-esophageal fistulization has developed, as reflected in the consistently high mortality reported in the literature [21,23].

## Conclusions

AEN secondary to thoracic aortic disease is a rare but life-threatening complication with high morbidity and mortality. Unlike the classical ischemic form, esophageal injury in this setting may result from direct mediastinal compression and compromised local vascular supply. This case emphasizes the diagnostic challenge of aortic-related esophageal ischemia, as dysphagia may precede overt necrosis or perforation by several weeks. Progressive dysphagia, persistent inflammation, gastrointestinal bleeding, or unexplained clinical deterioration after thoracic aortic rupture, dissection, or TEVAR should raise suspicion for esophageal involvement. Once transmural necrosis or fistulization develops, management must be individualized according to the extent of mediastinal contamination and the patient's physiological reserve. Early recognition and multidisciplinary management remain essential to improve outcomes in this uncommon but devastating complication. 

## References

[1] Gurvits GE, Shapsis A, Lau N, Gualtieri N, Robilotti JG (2007). Acute esophageal necrosis: a rare syndrome. J Gastroenterol.

[2] Sheikh AB, Mirza S, Abbas R, Javed N, Nguyen A, Hanif H, Farooq A (2022). Acute esophageal necrosis: an in-depth review of pathogenesis, diagnosis and management. J Community Hosp Intern Med Perspect.

[3] Abdul Haziz SR, Bickle I, Chong VH (2015). Dysphagia aortica: a rare cause of dysphagia. BMJ Case Rep.

[4] Papakonstantinou NA, Patris V, Antonopoulos CN, Samiotis I, Argiriou M (2019). Oesophageal necrosis after thoracic endovascular aortic repair: a minimally invasive endovascular approach-a dramatic complication. Interact Cardiovasc Thorac Surg.

[5] De Praetere H, Lerut P, Johan M (2010). Esophageal necrosis after endoprosthesis for ruptured thoracoabdominal aneurysm type I: can long-segment stent grafting of the thoracoabdominal aorta induce transmural necrosis?. Ann Vasc Surg.

[6] Chacón CP, Boix SC, Conde NR, Madina AG, Jofra M, Sala DG, Carrasco MA (2022). Esophageal perforation due to acute esophageal necrosis: a case report and a comprehensive literature review. J Clin Invest Surg.

[7] Koizumi S, Yamaguchi S, Asano S, Fujita H, Sueta T, Takeuchi S (2017). Esophageal necrosis after endovascular repair for ruptured aortic dissection. Asian Cardiovasc Thorac Ann.

[8] Watanabe S, Nagashima R, Shimazaki Y (1999). Esophageal necrosis and bleeding gastric ulcer secondary to ruptured thoracic aortic aneurysm. Gastrointest Endosc.

[9] Minatoya K, Okita Y, Tagusari O, Imakita M, Yutani C, Kitamura S (2000). Transmural necrosis of the esophagus secondary to acute aortic dissection. Ann Thorac Surg.

[10] Park NH, Kim JH, Choi DY (2004). Ischemic esophageal necrosis secondary to traumatic aortic transection. Ann Thorac Surg.

[11] Kaneda T, Onoe M, Asai T, Mohri Y, Saga T (2005). Delayed esophageal necrosis and perforation secondary to thoracic aortic rupture: a case report and review of the literature. Thorac Cardiovasc Surg.

[12] Porcu P, Chavanon O, Sessa C, Thony F, Aubert A, Blin D (2005). Esophageal fistula after endovascular treatment in a type B aortic dissection of the descending thoracic aorta. J Vasc Surg.

[13] Rascanu C, Weis-Müller BT, Fürst G, Grotemeyer D, Sandmann W (2009). Esophageal necrosis following endovascular treatment of a ruptured thoracal aortic aneurism: caused by mediastinal compartment syndrome (Article in German). Chirurg.

[14] Seto T, Fukui D, Tanaka H (2014). Tracheo-bronchial obstruction and esophageal perforation after TEVAR for thoracic aortic rupture. Ann Vasc Dis.

[15] Tobisch A, Ittrich H, Izbicki JR, Koenig AM (2014). Successful management of esophageal necrosis after endovascular repair of chronic type B aortic dissection. Ann Thorac Surg.

[16] Iuamoto LR, Chikami FH, Imakuma ES, Collet e Silva FS (2015). Acute esophageal necrosis secondary to stent: case report of the first surviving patient (Article in Portuguese). Rev Med.

[17] Abou-Al-Shaar H, Zaza KJ, Sharif MA, Koussayer S (2016). Free esophageal perforation following hybrid visceral debranching and distal endograft extension to repair a ruptured thoracoabdominal aortic aneurysm. Vasc Endovascular Surg.

[18] Joubert KD, Betzold RD, Steliga MA (2016). Successful treatment of esophageal necrosis secondary to acute type B aortic dissection. Ann Thorac Surg.

[19] Markar SR, Mackenzie H, Wiggins T, Askari A, Faiz O, Zaninotto G, Hanna GB (2015). Management and outcomes of esophageal perforation: a national study of 2,564 patients in England. Am J Gastroenterol.

[20] Lee KR, Stark E, Shaw FE (1977). Esophageal infarction complicating spontaneous rupture of the thoracic aorta. JAMA.

[21] Nachira D, Calabrese G, Senatore A (2024). How to preserve the native or reconstructed esophagus after perforations or postoperative leaks: A multidisciplinary 15-year experience. World J Gastrointest Surg.

[22] Fukunaga N, Shimoji A, Maeda T, Mori O, Yoshizawa K, Minatoya K, Tamura N (2023). Esophageal reconstruction as the first step for treating secondary aortoesophageal fistula due to thoracic endovascular aortic stent infection. Gen Thorac Cardiovasc Surg Cases.

[23] Rohatgi A, Papanikitas J, Sutcliffe R, Forshaw M, Mason R (2009). The role of oesophageal diversion and exclusion in the management of oesophageal perforations. Int J Surg.

